# Decellularized human liver as a natural 3D-scaffold for liver bioengineering and transplantation

**DOI:** 10.1038/srep13079

**Published:** 2015-08-07

**Authors:** Giuseppe Mazza, Krista Rombouts, Andrew Rennie Hall, Luca Urbani, Tu Vinh Luong, Walid Al-Akkad, Lisa Longato, David Brown, Panagiotis Maghsoudlou, Amar P. Dhillon, Barry Fuller, Brian Davidson, Kevin Moore, Dipok Dhar, Paolo De Coppi, Massimo Malago, Massimo Pinzani

**Affiliations:** 1 UCL, Institute for Liver and Digestive Health, University College London, London UK; 2 Stem Cells and Regenerative Medicine Section, Developmental Biology and Cancer Programme, UCL Institute for Child Health, Great Ormond Street Hospital. University College London, London UK; 3 Department of Cellular Pathology, UCL Medical School, Royal Free Campus, London UK; 4 Division of Surgery, Royal Free London Foundation Trust. University College London, London UK

## Abstract

Liver synthetic and metabolic function can only be optimised by the growth of cells within a supportive liver matrix. This can be achieved by the utilisation of decellularised human liver tissue. Here we demonstrate complete decellularization of whole human liver and lobes to form an extracellular matrix scaffold with a preserved architecture. Decellularized human liver cubic scaffolds were repopulated for up to 21 days using human cell lines hepatic stellate cells (LX2), hepatocellular carcinoma (Sk-Hep-1) and hepatoblastoma (HepG2), with excellent viability, motility and proliferation and remodelling of the extracellular matrix. Biocompatibility was demonstrated by either omental or subcutaneous xenotransplantation of liver scaffold cubes (5 × 5 × 5 mm) into immune competent mice resulting in absent foreign body responses. We demonstrate decellularization of human liver and repopulation with derived human liver cells. This is a key advance in bioartificial liver development.

Deaths from liver disease are increasing worldwide. According to the World Health Organisation, the total deaths caused by cirrhosis and liver cancer have increased by 50 million/year since 1990^1^. In the UK, the number of deaths from cirrhosis in those <65 years have increased ~6 fold in the last 30 years^2^. At present, liver transplantation is the only successful treatment for patients with end stage liver disease. However, 20% of patients die on the waiting list due to a shortage of organ donors^3^. To expand the supply of livers available for transplantation, transplant surgeons and physicians have explored several new approaches including split liver transplants, living-related partial donor procedures^4^ and the increasing use of “marginal” organs such as older donors, steatotic livers, non-heart-beating donors, donors with viral hepatitis, and donors with non-metastatic malignancy^5^. Despite these medical and surgical developments, it is unlikely that the availability of good liver grafts will ever be sufficient to meet the increasing demand of patients with end stage liver disease.

Alternatives to liver transplantation such as liver support systems, including bioartificial livers, and hepatocyte transplantation have been extensively explored but none adopted in clinical practice^6^,^7^,^8^,^9^,^10^,^11^.

In the UK, over 40% of the livers offered for transplantation are declined because of prolonged ischemic time or co-morbidities judged beyond marginal criteria^12^. This provides us with a major opportunity to explore alternative uses of human livers found to be unsuitable for transplantation following organ retrieval. In particular, while cellular viability is easily compromised, extracellular matrix (ECM) is better maintained in the discarded livers and it may be used as scaffold in which to grow normal human liver cells and recreate functional human liver tissue *in vitro*. Such cells could be obtained from recipient’s hepatocytes obtained from induced pluripotent stem (iPS) cells and thus avoid the need for immunosuppression. Alternatively, liver cells could be obtained from human livers unsuitable for liver transplantation or following liver resection. If successful, ECM liver bioengineering has the potential to be used not only as an alternative to whole organ transplantation but also for toxicity testing during preclinical drug development^13^ and for more stringent liver disease modelling.

Several studies have reported the decellularization of liver tissue obtained from animals^14^,^15^,^16^,^17^. The resulting 3-dimensional ECM scaffolds have been shown to provide an excellent environment for the *in vitro* growth of multiple liver cell types retaining excellent functionality^18^,^19^. Notably, in 2010, the repopulation of an acellular rat liver scaffold with 50 million mature rat hepatocytes was achieved by cell perfusion via the portal vein. Importantly, hepatocytes migrated beyond the matrix barrier to reach the decellularized sinusoidal spaces^20^. In 2012, a further step onward was made with the repopulation of a pig liver scaffold with human foetal hepatocytes and stem cells^21^. However, the decellularization and repopulation of a human liver ECM scaffold with human derived liver cells has not been reported.

The aim of this study was firstly to demonstrate the feasibility of an innovative protocol for the decellularization of a single lobe or of the whole human liver and to fully assess the quality and the *in vivo* biocompatibility of the resulting liver ECM scaffold. Secondly, to assess the capability of different types of human liver cells to repopulate hepatic ECM scaffolds (Fig. 1). Altogether the results of this study provide clear proof of concept data supporting the development of a bio-artificial liver tissue by employing decellularized human ECM liver scaffolds, thus opening, in general, more new possibilities in regenerative medicine for the use of donor human livers currently unsuitable for transplantation.

## Results

### Decellularization of human segmental lobes or whole liver

**Figure 2:**
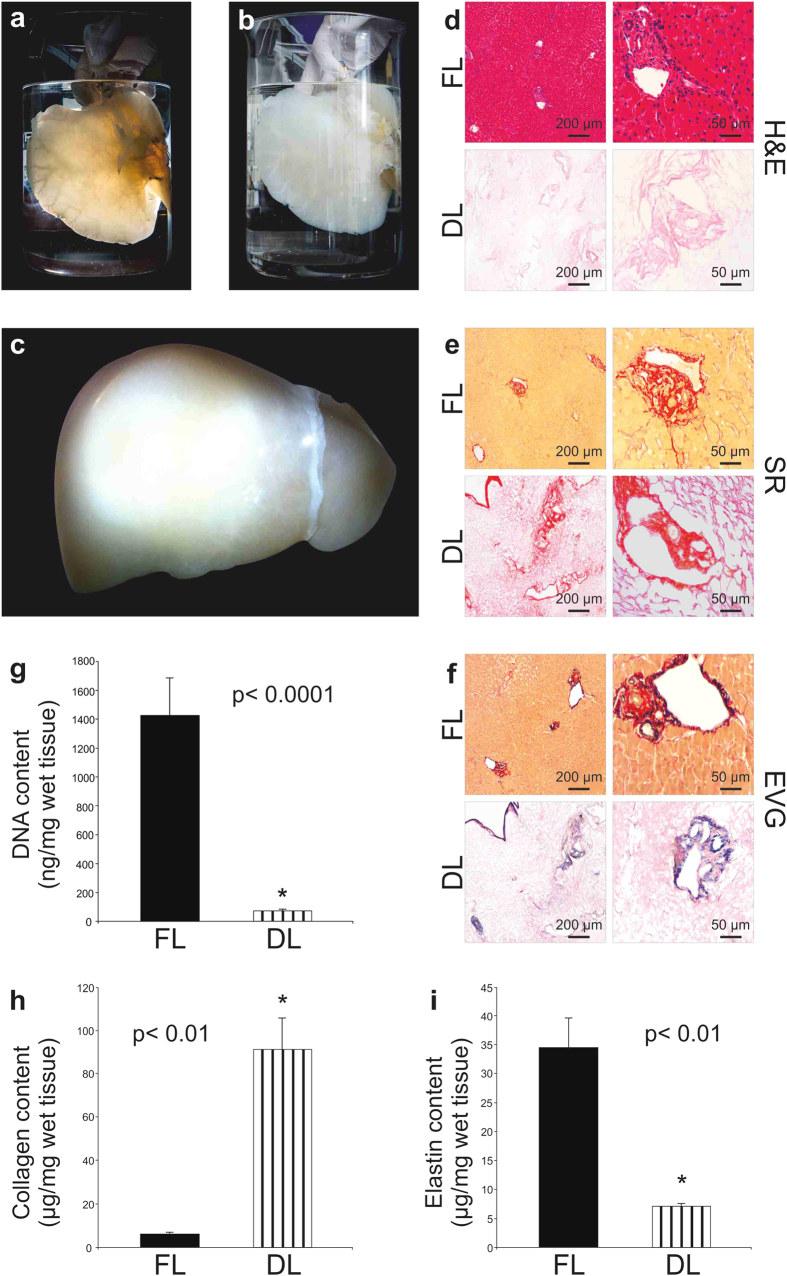
Perfusion-decellularization of human liver. Macroscopic appearance of a decellularized left lobe showing preservation of the vascular tree (**a**), translucent color (**b**) and a complete human liver (**c**). Histological comparison (10X and 40X magnification, left panel and right panel, respectively) of fresh liver (FL) and decellularized liver (DL) by Hematoxylin and Eosin (H&E) (**d**), Sirius Red (SR) (**e**) and Elastin Von Gieson EVG (**f**) staining demonstrating removal of cells and preservation of collagen and elastin in DL. Scale bar for 10X magnification: 200 μm and 40X: 50 μm. DNA quantification demonstrated significant DNA reduction from 1425.23 ± 261.37 ng/mg in FL to 47.91 ± 5.82 ng/mg in DL (**g**). Collagen significantly increased from 5.860726 ± 1.417547 μg/mg in FL to 90.85345 ± 14.16523 μg/mg in DL scaffolds (**h**). Elastin quantification demonstrated a significant decrease from 34.56827 ± 5.102387 μg/mg to 7.073619 ± 0.434233 μg/mg in in DL scaffolds (**i**).

Decellularization of the left lobe of the liver (segments 1-2-3) was completed within 14 days of perfusion, and within 6 weeks for the whole human liver (Fig. 2a–c). During and following decellularization the whole liver or lobes gradually became increasingly translucent with the dissolution of cells (Fig. 2a–c). The decellularization protocol, based on a retrograde perfusion through the hepatic venous system and inferior vena cava, was characterized by the combination of five different Cell-Damaging Factors (5CDFs): i) mechanical cell damaging (freezing/thawing) to favor cell destruction; ii) isotonic stress to allow cell lysis; iii) enzymes to allow cell detachment; iv) action of detergents to remove debris; and v) flow shear stress to allow the penetration into the hepatic sinusoid leading to the detachment of cells and debris. Histological evaluation by H&E staining showed no evidence of cell bodies or cellular material including nuclei in the decellularized liver (Fig. 2d). In addition, the general liver tissue architecture appeared fully preserved as shown by Sirius Red staining for collagens (Fig. 2e) and Von Gieson staining for elastin (Fig. 2f).

The absence of cells in the ECM scaffold was confirmed by the absence of DNA material quantified by DNeasy Blood and Tissue kit (Fig. 2g). As already reported in other perfusion-decellularization protocols^17^,^22^,^23^, collagen content relative to wet weight was increased (p < 0.01; Fig. 2h), whereas elastin was decreased (p < 0.01) in the decellularized tissue when compared with fresh liver tissue (Fig. 2i).

### Liver tissue scaffold characterisation

**Figure 3:**
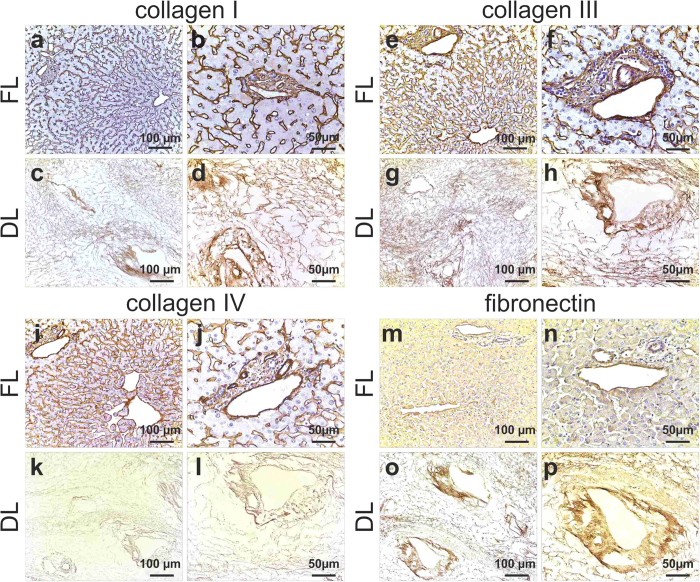
Expression and distribution of ECM proteins. Collagen I, III and IV staining in FL is seen as fine strands in the parenchymal space as well as around the blood vessels (**a**,**b**; **e**,**f**; **I**,**j**). Collagen I and III distribution was preserved following decellularization as demonstrated by a staining in both sinusoids and portal tracts in DL (**c**,**d**; **g**,**h**). Collagen IV (**k**,**l**) and fibronectin (**o**,**p**) staining showed a conserved meshwork in sinusoids and biliary ducts after decellularization. Scale bar for 20X magnification (left panel): 100 μm and 40X (right panel): 50 μm.

The expression and distribution of the extracellular matrix (ECM) of the decellularized liver (DL) scaffold was next analysed, comparing it with ECM of fresh human liver tissue (FL) by immunohistochemistry. This analysis showed that the expression and distribution of key ECM components, namely collagen type I (Fig. 3c,d), collagen type III (Fig. 3g,h), fibronectin (Fig. 3o,p) and collagen IV (Fig. 3k,l) were fully maintained when compared to that detected in FL. Importantly, the pattern of distribution of each ECM component within the DL confirmed the full preservation of the architectural organisation of the original liver tissue (FL).

### Ultrastructural characterization of decellularized human liver

**Figure 4:**
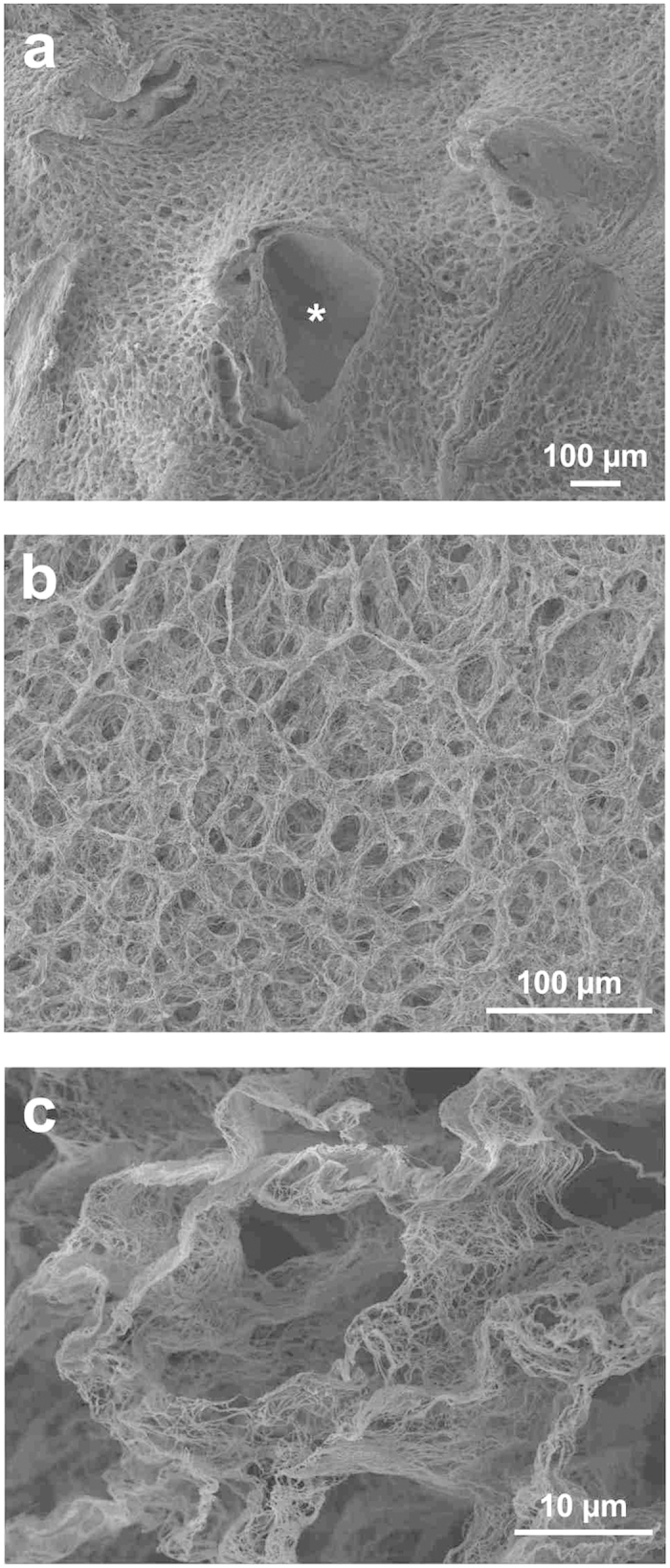
Ultrastructural characterisation of decellularized livers. Low magnification SEM image confirmed acellularity in the scaffolds and demonstrated preservation of the three-dimensional micro-anatomy of the portal tract (arrow/asterisk) surrounded by a typical lobular structure (**a**). The parenchymal space appears characterised by connective tissue fibres arranged with a honeycomb-like pattern, clearly defining hepatocyte-free spaces (**b**). High magnification resolution demonstrates an exceptionally preserved three-dimensional meshwork of connective tissue fibres structuring the hepatocyte-free spaces (**c**). Scale bars for (**a**,**b**): 100 μm and (**c**): 10 μm.

Scanning electron microscopy was used to evaluate the impact of decellularization on the 3D-architecture and micro-structure of the ECM (Fig. 4). The overall liver tissue micro-structure appeared maintained. Key architectural structures such as portal tracts were clearly recognised with their typical features and distribution within the scaffold (Fig. 4a, asterisk). The areas corresponding to the liver lobule were characterised by a three-dimensional network of connective tissue fibres arranged in a honeycomb-like structure (Fig. 4b). Most notably, the hepatocyte pocket was preserved and was surrounded by a thorough network of ECM proteins (Fig. 4c). Overall, these data confirm the preservation of the 3D liver microanatomy and ultrastructure following decellularization.

### Interspecies bio-compatibility of decellularized human liver ECM scaffolds

**Figure 5:**
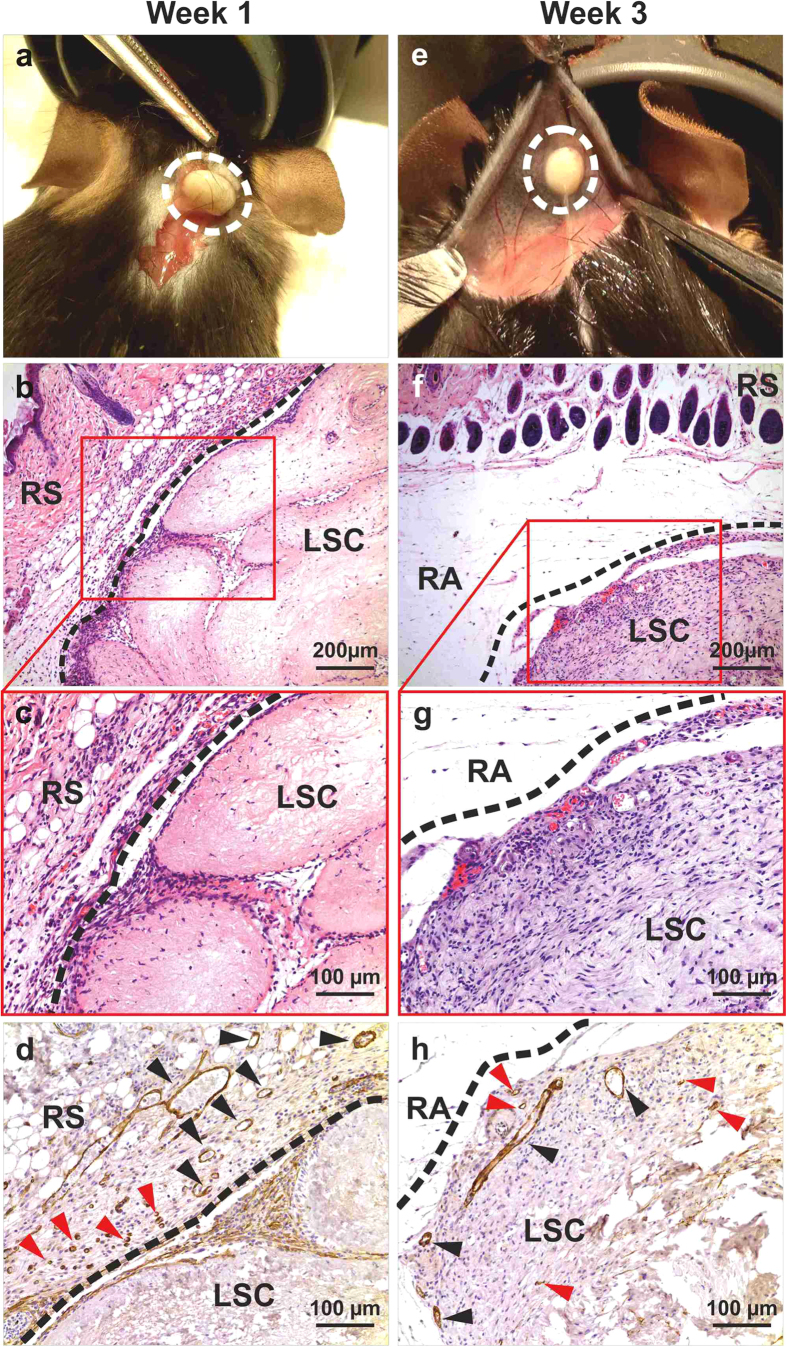
Interspecies biocompatibility: subcutaneous implantation. Human liver cubic scaffolds were implanted subcutaneously (n = 6) and evaluated at 7 and 21 days post-implantation. Polymorphonuclear cells and lymphocytes were observed at 7 days post-implantation, indicating a mild inflammatory response. Inflammatory cells were mostly seen in the tissue around the implants (**b**,**c**). By contrast, at 21 days post-implantation, little or no inflammatory infiltrate was observed around the implants (panels **f**,**g**). Immunohistochemistry for alpha-SMA showed SMA-negative, spindle-like cells, had infiltrated the implants after 21 days post-implantation (**h**) and revealed the presence of abundant neo-vessels (mostly arterioles) initially close to the interface host tissue/human scaffold (7 days, **d**, arrows) and subsequently deeper within the scaffold (21 days, **h**, arrows) indicating neovascularisation of the implant. Scale bars: for 10X magnification (**b**,**f**): 200 μm, 20X (**c**,**d**,**g**,**h**): 100 μm. Abbreviations: RS (recipient skin), LSC (liver scaffold) and RA (recipient adipose tissue).

**Figure 6:**
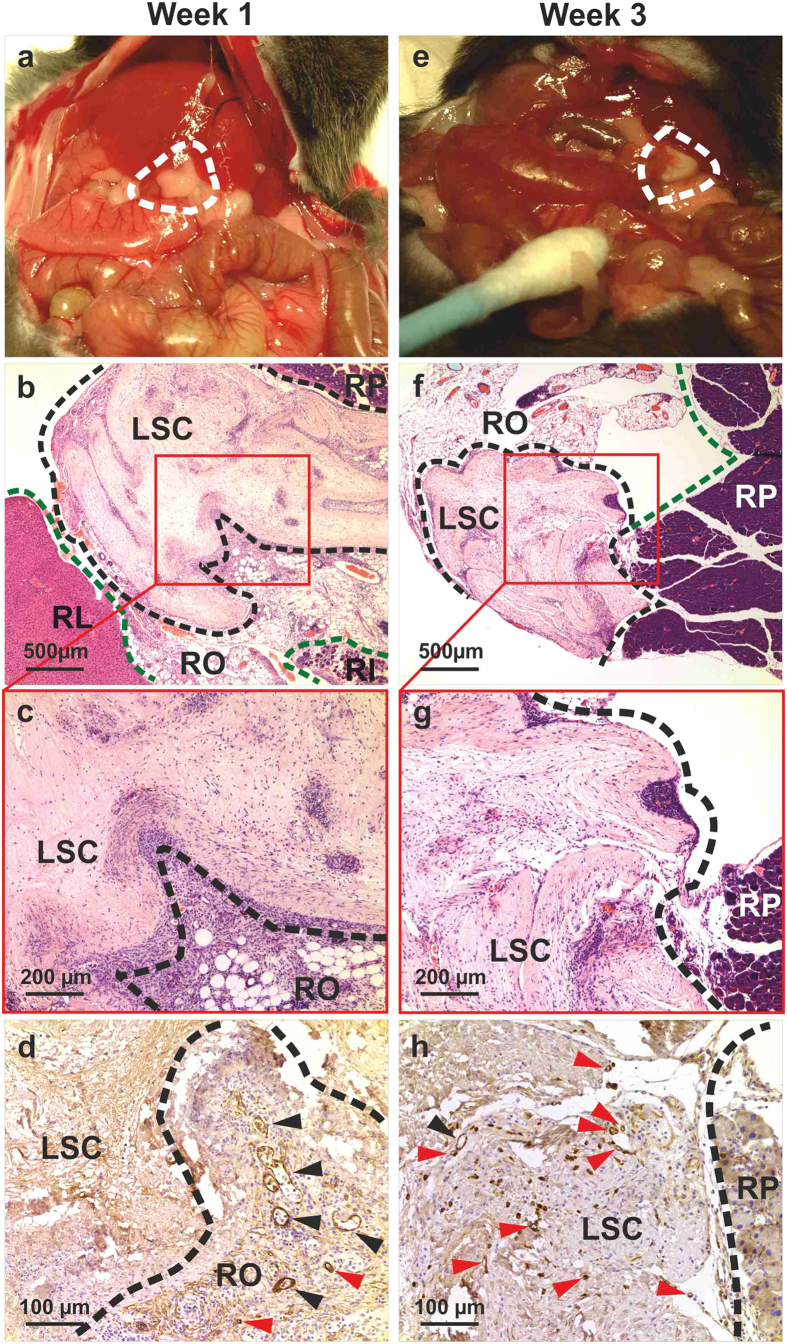
Interspecies biocompatibility: omental implantation. Human liver cubic scaffolds were implanted into the omentum (n = 6), and evaluated at 7 and 21 days post-implantation. As early as seven days after implantation, the implanted scaffold engrafted within the surrounding host abdominal structures: recipient liver (RL), recipient intestine (RI) and recipient pancreas (RP). Polymorphonuclear cells and lymphocytes were observed at 7 days post-implantation, indicating a mild inflammatory response. Inflammatory cells were mostly seen in the tissue around the implants (**b**,**c**). By contrast, at 21 days post-implantation, little or no inflammatory infiltrate was observed around the implants (panels **f**,**g**). Immunohistochemistry for alpha-SMA showed SMA-negative, spindle-like cells, had infiltrated the implants after 21 days post-implantation (**h**) and revealed the presence of abundant neo-vessels (mostly arterioles) initially close to the interface host tissue/human scaffold (7 days, **d**, arrows) and subsequently deeper within the scaffold (21 days, h, arrows) indicating neovascularisation of the implants. Scale bars: for 4X magnification (**b**,**f**): 500 μm, 10X (**c**,**g**): 200 μm and 20X (**d**,**h**): 100 μm. Abbreviations: RL (recipient liver), RP (recipient pancreas), RI (recipient intestine), RO (recipient omentum) and LSC (liver scaffold).

Cubic fragments of ECM scaffold (5 × 5× 5 mm^3^ for a total volume of 125 mm^3^) were obtained by scalpel cleavage of the whole decellularized livers, and implanted into immunocompetent mice to evaluate the *in vivo* bio-compatibility in a xenotransplantation model. Liver ECM cubes were implanted either subcutaneously (n = 6) or into the omentum (n = 6), and evaluated at 7 and 21 days post-implantation. Clinical behaviour of animals and local signs of inflammation were closely monitored. Both subcutaneous and omental implanted liver scaffolds were evaluated macroscopically, histologically and immunohistochemically for the presence of inflammatory reaction, foreign body responses and angiogenesis. During the period of observation, mice showed normal behaviour, without local signs of inflammation, implant exposure, extrusion or death. Macroscopic examination at the time of enucleation showed healthy normal tissue around the implants, with no signs of inflammation, such as redness and swelling, or adverse tissue reactions around the implants, such as destruction of normal structures, at both subcutaneous and omental sites (Figs 5 and 6, panels a,e, respectively).

Histology was performed on formalin-fixed samples embedded in paraffin and sectioned at 4- μm thickness. Representative sections were stained with haematoxylin and eosin and immunostained for alpha smooth muscle actin (alpha-SMA) and CD3. Histological analysis confirmed the absence of a foreign body reaction, and the absence of giant cells or granulomata within any of the samples. Polymorphonuclear cells and lymphocytes were observed at 7 days post-implantation, indicating a mild inflammatory response surrounding the implantation sites. Inflammatory cells were mostly seen in the tissue around the implants (Figs 5 and 6, panels b,c). Immunohistochemistry indicated that infiltrating host cells were predominantly CD3+ T-cell lymphocytes (data not shown). By contrast, at 21 days post-implantation, reduced or no inflammatory infiltrate was observed around the implants (Figs 5 and 6, panels f,g).

Immunohistochemistry for alpha-SMA revealed that alpha-SMA-negative, spindle-like cells, most likely representing fibroblasts, had infiltrated the implants after 21 days post-implantation (Figs 5 and 6, panel h). On the other hand, positivity for alpha-SMA revealed the presence of abundant neo-vessels (mostly arterioles), initially close to the interface host tissue/human scaffold (7 days, Figs 5 and 6, panel d) and subsequently deeper within the scaffold (21 days, Figs 5 and 6, panel h), thus providing evidence for neovascularisation of the implant.

### Re-population of decellularized human liver ECM scaffolds with human liver cell types

**Figure 7:**
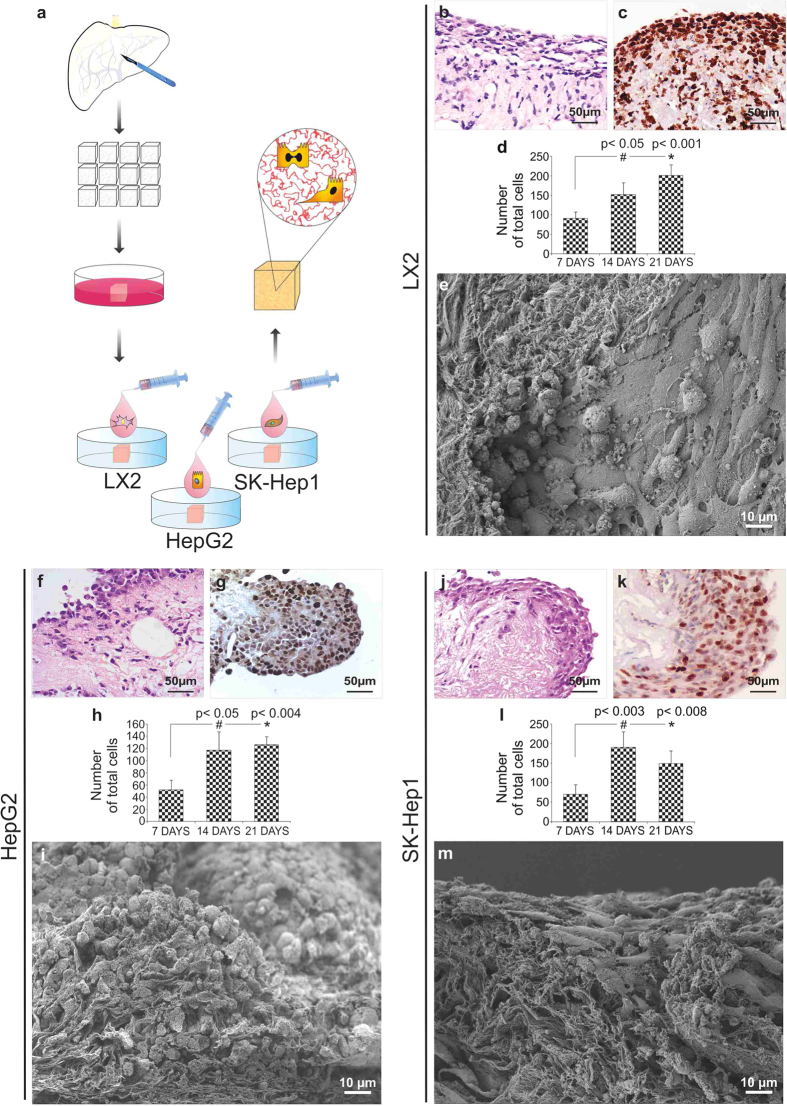
Recellularisation of human liver scaffolds. Human liver cubic scaffolds were incubated in complete medium overnight before seeding the scaffolds with LX2, HepG2 and Sk-Hep-1 cell lines (**a**). Bioengineered liver tissues were harvested at 7, 14 and 21 days. H&E (**b**,**f**,**j**) and Ki67 (**c**,**g**,**k**) staining showed that all cell types were able to repopulate liver scaffolds while still proliferating at 21 days. The total cell count in human liver scaffolds repopulated with LX2, HepG2 and Sk-Hep-1, increased significantly between 7 and 14–21 days (**d**,**h**,**l**, respectively). Scale bar for 40X magnification (H&E and KI67): 50 μm. SEM (**e**) LX2 cells migrated within the decellularized sinusoidal space acquiring different morphologies: from a migratory flattened fibroblast-like cell phenotype, to a rounded shape; (**i**) HepG2 cells, showing an epithelioid phenotype, were spread diffusely and engrafted into the ECM scaffold; (**m**) Sk-HEP-1 cells repopulated the scaffold showing a mesenchymal-like phenotype. Scale bar SEM images = 10 μm.

Human liver ECM cubes were incubated in complete medium overnight before seeding the scaffolds with LX2 (a human hepatic stellate cell line), HepG2 (epithelial cells derived from hepatocellular carcinoma) or Sk-Hep-1 (endothelial cells, derived from highly invasive human adenocarcinoma) human cell types (Fig. 7a). Cells [2 × 10^6^/50 μL] were drawn up in a 0.5 ml insulin syringe and released drop by drop to finally cover the liver ECM cubes. Cubes incubated with the different cell types were evaluated after 7, 14 and 21 days of *in vitro* static culture. H&E staining demonstrated the progressive engraftment of LX2 cells into the scaffold over 21 days (Fig. 7b) with a diffuse presence of proliferative cells (Fig. 7c). Indeed, the number of cells increased significantly (Fig. 7d) between 7, 14 and 21 days of culture (p < 0.05 and p < 0.001 at 14 days and 21 days, respectively). Scanning electron microscopy (SEM; Fig. 7e) further demonstrated that LX2 cells migrated within the decellularized sinusoidal space acquiring different morphologies: from a flattened fibroblast-like cell phenotype, likely migratory, to a round-up shape probably indicating mitosis. Liver ECM cubes were also successfully repopulated with HepG2 cells, which rapidly engrafted and migrated through the scaffold (Fig. 7f). Repopulation with HepG2 cells was also characterized by marked Ki67 positivity indicating cells proliferation (Fig. 7g). Accordingly, there was a marked increase in cell number between 7, 14 and 21 days (Fig. 7h; p < 0.05 and p < 0.004 at 14 days and 21 days, respectively). SEM showed that HepG2 cells, showing an epithelioid phenotype, were spread diffusely and engrafted into the ECM scaffold (Fig. 7i).

Metastatic Sk-Hep-1 cells rapidly repopulated the liver ECM cubes (Fig. 7j) showing a high proliferation rate (Fig. 7k) and a remarkable increase in total cell number between 7, 14 and 21 days (Fig. 7l; p < 0.003 and p < 0.008 at 14 and 21 days, respectively). Moreover, SEM images demonstrated that Sk-Hep-1 cells aggressively repopulated the scaffold showing a mesenchymal-like phenotype (Fig. 7m).

## Discussion

The present paper describes our experience on the decellularization of human liver to generate human liver scaffolds. These results offer a key advance towards the use of healthy human livers unsuitable for liver transplantation for tissue engineering and ultimately aimed at functional organ replacement, disease modelling and assessment of drug efficacy/toxicity.

The first successful decellularization of a liver tissue, i.e. porcine, was reported in 2004 by Lin and Colleagues^24^. Repopulation of this scaffold with primary rat hepatocytes revealed a significantly higher albumin and urea synthesis by cells cultured within the scaffold when compared to monolayers of the primary hepatocytes cultured on collagen gels, thus suggesting a greater functional potential of cells cultured in a 3D scaffold. Since 2004, several other reports have described the successful decellularization of rat, rabbit or pig livers^14^,^15^,^16^,^17^. More recently, Baptista and Colleagues have demonstrated the repopulation of decellularized pig liver with human hepatocyte progenitor cells or with foetal hepatocytes and foetal hepatic stellate cells for up to 13 days. This system showed active metabolism and albumin synthesis^25^.

The extensive exploitation of pig livers is related to both their wide availability and dimensions in a range compatible with the size of human liver. However, the ultrastructure of porcine liver differs significantly from that of human liver. Indeed, pig liver has well defined lobules outlined by connective tissue (portal to portal lining), which is absent in healthy human liver but present in fibrotic liver^26^ and it may not represent the ideal ECM for human liver tissue engineering. Thus, given the wide availability of donor human livers unsuitable for liver transplantation, it would be beneficial to make a step towards the optimal use of these organs for regenerative medicine. This approach towards the development of human tissue ECM scaffolds has been already proposed for kidney^27^, heart^28^ and lung^29^ in recent years.

The first important contribution emerging from the work herein presented is the description of a novel decellularization protocol which was designed in order to resolve many of the hurdles described in previously published protocols, with a special focus on the structure and biochemical features of human liver tissue. In particular, the application of decellularization protocols previously reported for the decellularization of non-human livers and characterised by constant flow rate and anterograde perfusion^30^, repeatedly resulted in suboptimal results. Therefore, a novel protocol based on retrograde perfusion combined with two steps perfusion flow/rate was applied with the successful removal of immunogenic cellular materials, whilst maintaining the 3D architecture and essential extracellular matrix proteins. The resulting liver scaffold was robust, in that it could be easily handled without disintegration or rupture, maintaining its tissue flexibility.

The observation that collagen types I and IV, and fibronectin were present in bundles around the vascular and biliary structures, portal triads, and central veins, as well as in the space of Disse, emphasises the preservation of liver architecture. This was further confirmed by SEM showing an extremely well preserved 3D-microanatomy of the portal tracts and liver lobules, and demonstrated the conservation of a three-dimensional meshwork of connective tissue fibres around the hepatocyte-free spaces.

Biocompatibility is an important issue to be clarified when proposing bio-technologies potentially leading to Advanced Therapy Medicinal Products^31^ (ATMP). In the case of a 3D human ECM scaffold the question is whether or not the ECM proteins present in the scaffold are able to evoke an immune response and rejection in non-matched recipient thus requiring continued immunosuppression^32^. The results obtained by implanting cubic fragments of human liver scaffold subcutaneously or intra-abdominally in immune competent mice show that there was no discernible immune reaction or rejection of the scaffold. The implanted scaffold appeared to be incorporated into the host tissues with progressive host cell infiltration and arteriolar neovascularisation in both omental and subcutaneous implantations. This latter aspect is striking and deserves further investigation in order to define models of autologous repopulation by host-derived endothelial and other mesenchymal cells following implantation of a biological scaffold. Indeed, the model of re-creating autologous, i.e. host-derived, liver stroma, by implantation of a 3D ECM scaffold repopulated with functional hepatocytes derived from different potential sources, (e.g stem cells and iPSCs) represents an attractive line of research.

Finally, the repopulation of the ECM scaffold with different types of liver cells and the confirmation that the 3D structure enables and allows the efficient homing and targeting of cells to their correct location is an indispensable step in the characterisation of the possible use of the ECM scaffolds for both regenerative medicine and disease modelling. To explore this aspect, three different cells lines were selected as representative prototypes to test scaffold repopulation: human hepatic stellate cells (LX2 cells) as typical liver-specific pericytes and main effectors of hepatic fibrogenesis^33^, HepG2^34^ cells as well differentiated neoplastic cells reproducing many of the features of normal hepatocytes, and Sk-Hep-1^35^ cells, reproducing the features of an invasive metastatic adenocarcinoma. All three different cell types engrafted without difficulty into the cubic scaffold fragments with successful migration and cellular proliferation, although with different cell type-dependent dynamic and topographic features. These data confirm that the human liver bio-scaffold retains the essential characteristics necessary to provide homing of the cells to specific locations inside the scaffold.

Altogether the current results provide an innovative basis for further developments in the area of hepatic regenerative medicine through the use of human deceased donor livers that are unsuitable for whole organ transplantation. On this basis, the way to progress towards potential clinical applications should be directed at: a) the evaluation of different protocols for recellularization by employing perfusion bio-reactors allowing optimal cell homing when different cell types are employed, either singularly or together; b) proteomic studies to assess the preservation of ECM proteins and the presence of residual cellular materials; c) the assessment of the metabolic requirements and functionality of the bioengineered liver, and d) an estimation of the accuracy of different types of cell repopulation in reproducing 3D scenarios of liver pathophysiology fundamental for liver disease modelling as well as for drug toxicity/efficacy testing. In this context, a key challenge will be represented by the re-endothelization of hepatic sinusoids and portal triad vasculature.

## Methods

### Source of human livers

The work described in this paper was performed on healthy human livers that were harvested for transplantation and then judged unsuitable because of prolonged graft cold ischaemic time, the presence of extra-hepatic malignancy or other important extra-hepatic co-morbidities in donors or recipients. Livers included in this study were defined “healthy” because of the absence of any degree of tissue fibrosis and fat accumulation by histological analysis. The study was approved by the UCL Royal Free BiobankEthical Review Committee (NRES Rec Reference: 11/WA/0077). Informed consent for research was confirmed via the NHSBT ODT organ retrieval pathway, and the project was also approved by the NHSBT Research Governance Committee. Donor livers were processed in accordance with the UCL Royal Free Biobank protocols under the Research Tissue Bank Human Tissue Act licence, prior to use in research. Human livers obtained at the Royal Free London Foundation Trust were coordinated, received and recorded by the UCL Tissue Access for Patient Benefit organisation (TAPb) which links research activities between UCL Royal Free Biobank, the Royal Free Trust and UCL. TAPb has full governance in place for this purpose which has involved NHSBT ODT pathway, the Human Tissue Authority licencing, and the local Trust/UCL Research offices.

### Surgery of human liver in preparation for the decellularization procedure

Human livers (n = 3) were either surgically processed in order to obtain the liver left lobes (n = 2) [374 g and 250 g, respectively] including segments 1, 2, 3 and 4 or retained as a whole liver (n = 1) [1, 774 Kg] with preserved vascular access.

Whole liver preparation: the donor liver was processed exactly as for liver transplantation; thereafter the upper caval cuff, reaching the atrial rim, was oversewn with a running double row of 3-0 prolene suture. The water tightness of the organ was proofed both by antegrade portal and retrograde hepatic venous perfusion under relative high pressure as produced by a 50 ml bladder syringe with PBS solution.

Segmental liver preparation of left lateral liver (S1 + S2 + S3 + S4): dissection is started at the hilar region with inspection, preparation, division and ligation of the hepatic artery (HA), of the portal vein (PV) and of the bile duct (BD). Segment 4 HA is preserved if originating from the right hepatic artery, forcing the division of the right HA distal to its bifurcation. The right hepatic vein is divided and the middle and left hepatic veins are preserved with the future left reduced size left liver. The line of parenchymal division is maintained to the left of the line of Cantlie in order to maintain the left portion of S4. The parenchyma is divided sharply with a knife for the liver capsule and subsequently with crush-clamp technique, performing meticulous hemo- and bilio-stasis. The parenchymal division is carried to the end, producing a left lateral liver extended to S4 and a right liver as non-functional unit. Finally, the upper caval cuff, reaching the atrial rim is oversewn with a running double run of 3-0 prolene suture.

Human livers were then frozen at −80 °C for at least 24 h for the purposes of initial destruction of the various cellular compartments.

### Decellularization Protocols of Whole Human Liver Lobe

The perfusion regime for the decellularization of the liver left lobe is shown in Table1. The decellularization of the whole liver was achieved by repeating the procedure shown in Table 1 three times (the freezing/thawing step was performed just once). The following abbreviations were used: distilled Water (dH_2_O), TX100 (Triton X100), SDS (sodium dodecyl sulfate), A-A 5% (Antibiotic and Antimycotic), PAA (paracetic acid) and EtOH (ethanol) purchased from Sigma Aldrich.

**Figure 8:**
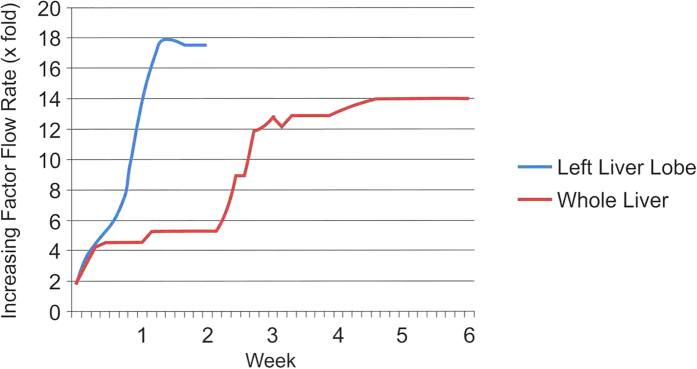
Flow rate. The graph illustrates the increasing flow rate (x fold) over time starting with an initial flow rate of 0.2–0.3 ml/min/g of liver. The decellularization of a human liver left lobe (blue line) was achieved in 2 weeks, while the decellularization of a whole human liver (red line) was completed in 6 weeks.

The initial flow rate for decellularization perfusion was 0.2–0.3 ml/min/g of liver. Subsequently, two phases of perfusion were adopted a) steeply increasing flow rate to compensate reduced resistance and b) stabilization of the flow rate as the decellularization proceeds. The two phases of flow rate are shown in Fig. 8. After decellularization, 125 mm^3^ cubic scaffold fragments (5 mm×5 mm×5 mm) were dissected by scalpel cleavage for further characterization and liver bioengineering *in vitro.*

### Histology and immunostaining analysis

Samples were fixed for at least 24 hours in 10% neutral buffered formalin solution (pH 7.4) at RT. Tissue was embedded in paraffin and sectioned at 4 μm. Prior to staining sections were dewaxed in xylene and rehydrated using graded industrial denatured alcohol (IDA).

Histochemical stains: tissue sections were stained with Harris’s Haematoxylin and Eosin (H&E) (Leica, Germany), Picro-Sirius Red (SR) (Hopkin & Williams) (BDH Chemicals Ltd, Cellpath Ltd) and stains Miller’s Elastic stain with a Picro-Sirius red counter stain (VWR, Leica, Raymond A Lamb).

Immunocytochemistry: sections stained with Collagen I, III, IV, fibronectin and laminin were incubated in 0.5% Trypsin (MP Biomedical)/0.5% Chymotrypsin (Sigma)/1% Calcium Chloride (BDH) in Tris buffered saline pH 7.6 (TBS) for 30 minutes at 37 °C. Sections stained with alpha-smooth muscle actin (SMA) were microwaved (640 W) for 20 minutes in 1L of Tris-EDTA buffer (10 mM Tris-base/1 mM EDTA solution, pH9.0) and sections for CD3 pressure cooked for 3 mins in sodium citrate buffer (10 mM Sodium Citrate, pH 6.0). Slides were then soaked in TBS with 0.04% Tween-20 (Sigma) for 5 mins, blocked in peroxidase blocking solution (Novocastra) for 5 minutes, washed in TBS for 5 mins and then incubated for 1 hour in the following primary antibodies; collagen I (Rabbit pAb to coll1 (ab34710), diluted 1:200; Abcam), collagen III (Rabbit pAB to coll3 (ab7778), diluted 1:500; Abcam), collagen IV (mouse mAb to coll4 (M0785), diluted 1:25; Dako), fibronectin (mouse mAb to fibronectin (MAB1937), diluted 1:100; Millipore), laminin (mouse mAb to laminin α5-chain (MAB1924), diluted 1:200; Millipore), alpha-Smooth Muscle Actin (mouse mAb to SMA, (M0851/1A4), diluted 1:500; Dako) and CD3 (rabbit pAb to CD3, (AO452), diluted 1:200; Dako). The slides were then placed for 25 minutes in NovolinkTM post primary (Novocastra), 25 mins in NovolinkTM polymer solution (Novocastra) and developed with NovolinkTM 3,3′ di-amino-benzidine (Novocastra) with a 5 minute wash in TBS with 0.04% Tween-20 between each step. Slides were counterstained with Mayer’s Haematoxylin (Sigma) for 3 min. All sections were dehydrated in graded IDA, cleared in xylene and were mounted with DPX (Leica biosystems); cover slipped and observed using a Zeiss Axioskop 40. Images were captured with an Axiocam IcC5 using Zeiss Axiovision (verison 4.8.2). All images were analysed and enhanced using Fiji v1.49d (ImageJ Jenkins server).

### DNA quantification

To assess total DNA content within native tissue and acellular matrices, the DNeasy Blood and Tissue kit was used according to the manufacturer’s manual (Qiagen). Briefly, specimens were digested with Proteinase K overnight. DNA samples were purified using buffers provided by the company and measured spectrophotometrically (Nanodrop, Thermo Scientific, US). Optical densities at 260 nm and 280 nm were used to estimate the purity and yield of nucleic acids.

### Collagen quantification

The collagen content of native tissue and decellularized tissue was quantified using the total collagen assay kit according to the manufacturer’s manual (QuickZyme Biosciences, The Netherlands). Briefly, samples were hydrolysed in 6M HCl at 95 °C for 20 hours, the hydrolysates were mixed with a chromogen solution staining the hydroxyproline residues and color was developed at 60 °C for 1 hour. The absorbance for each sample was determined at 555 nm using a FLUOstar Omega microplate reader (BMG labtech, Germany) and the collagen quantity was calculated by usage of a standard curve of pure collagen hydrolysates.

### Elastin quantification

The elastin content of native and decellularized tissue was quantified using the FASTIN elastin assay (Biocolor, UK) according to the manufacturer’s instructions. Briefly, the samples were homogenized, and elastin was solubilized in 0.25 M oxalic acid. Two consecutive incubations were performed at 95 °C to ensure complete extraction of elastin. Extracts were incubated with 5,10,15,20-tetraphenyl-21H,23H-porphine tetrasulfonate (TPPS) dye, and absorbance was determined at 513 nm spectrophotometrically FLUOstar Omega microplate reader (BMG labtech, Germany). Elastin concentrations from a standard curve were used to calculate the elastin content of the tissue.

### Scanning Electron Microscopy (SEM)

Samples were fixed in 2.5% glutaraldehyde in 0.1 M phosphate buffer and left for 24 hours at 4 °C. Following washing with 0.1 M phosphate buffer, samples were cut into segments of approximately 1 cm length and cryoprotected in 25% sucrose, 10% glycerol in 0.05 M PBS (pH 7.4) for 2 hours, then fast frozen in Nitrogen slush and fractured at approximately −160 °C. Next, samples were then placed back into the cryoprotectant at room temperature and allowed to thaw. After washing in 0.1 M phosphate buffer (pH 7.4), the material was fixed in 1% OsO4 / 0.1 M phosphate buffer (pH 7.3) at 3 °C for 1½ hours and washed again in 0.1 M phosphate buffer (pH 7.4). After rinsing with dH2O, specimens were dehydrated in a graded ethanol-water series to 100% ethanol, critical point dried using CO_2_ and finally mounted on aluminum stubs using sticky carbon taps. The fractured material was mounted to present fractured surfaces across the parenchyma to the beam and coated with a thin layer of Au/Pd (approximately 2 nm thick) using a Gatan ion beam coater. Images were recorded with a 7401 FEG scanning electron microscope (Jeol, USA).

### Xenotransplantation in immunocompetent mice

All animal experiments were approved by the Home Office under the UK Animals and Scientific Procedures Act 1986 and in accordance with the guidelines of the Comparative Biology Unit, Biological Services University College London (UCL) under Project license 70/7100.

For biocompatibility studies, twelve male C57BL/6J mice, aged 3–4 weeks, were used. Human liver cubic scaffolds were surgically implanted either subcutaneously (n = 6) or into the omentum (n = 6). Following shaving the operating area, the skin was cleaned with 10% povidone iodine (Videne, Ecolab, Leeds, UK) and 20% chlorhexidine gluconate (Hydrex pink, Ecolab, Leeds, UK). Isoflurane (2% with 98% oxygen) was used to induce anesthesia. For subcutaneous implantation, a small incision (5 mm) was made between the shoulder blades and a subcutaneous tunnel was made by blunt dissection. The human liver cubic scaffold was inserted into the subcutaneous tunnel and the wound was closed with absorbable 4-0 vicryl sutures. When applying an omental implantation, a small midline abdominal incision was made and the human liver cubic scaffold was folded with the omentum and was secured in place by 6-0 vicryl sutures. The abdomen was closed in two layers. After 7 and 21 days, mice were euthanized and the implants along with the surrounding tissues were harvested and fixed in 10% formalin for histological and immunohistochemical evaluation.

### Cell Culture

The LX2 cell line is a well-established hepatic stellate cell line that was generated by a spontaneous immortalization in low serum conditions^30^. Cells are cultured in Iscove’s Modified DMEM supplemented with 2 mM/L glutamine, 0.1 mM/L non-essential amino acids, 1.0 mM/L sodium pyruvate and 20% Foetal Bovine Serum (FBS). HepG2 and Sk-Hep-1 cells (ATCC® HTB-52™) are derived from a human hepatoblastoma and a human hepatocellular carcinoma, respectively. Both cell types were purchased from ATCC (VA, USA) and cultured in Eagle’s Minimum Essential medium (EMEM), supplemented with Glutamax, 0.1 mM/L non-essential amino acids, 1.0 mM/L sodium pyruvate and 10% FBS. All cells were cultured under standard conditions in a humidified incubator under 5% CO_2_ and at 37 °C. Every 3 days the complete culture medium was changed and sub-confluent cells were trypsinized and passaged at a split ratio 1:3.

### Repopulation and culture of engineered human liver

Human liver cubic scaffolds were kept overnight in complete medium [day -1]. Cells were re-suspended at a concentration of 2 million cells per 50 μl (2 × 10^6^/50 μL) per scaffold (n ≥ 12 per cell line). Cells were drawn up in a 0.5 ml insulin syringe and released drop by drop to finally cover the decellularized tissue. Seeded scaffolds were kept for 2 h in a humidified environment at 37 °C with 5% CO_2_ allowing cell attachment followed by addition of complete culture medium [day 0]. The culture medium was changed at day 1 and afterwards every 3 days. At days 7, 14 and 21 following seeding, the scaffolds were placed in 10% formaldehyde and assessed by histology and immunohistochemistry or fixed in 2.5% glutaraldehyde for SEM analysis.

### Statistical analysis

Results were expressed as mean ± s.d. All data was analysed with ANOVA or Student’s t-test. Two-talied p values less than 0.05 were considered statistically significant.

## Additional Information

**How to cite this article**: Mazza, G. *et al.* Decellularized human liver as a natural 3d-scaffold for liver bioengineering and transplantation. *Sci. Rep.* **5**, 13079; doi: 10.1038/srep13079 (2015).

## Figures and Tables

**Figure 1:**
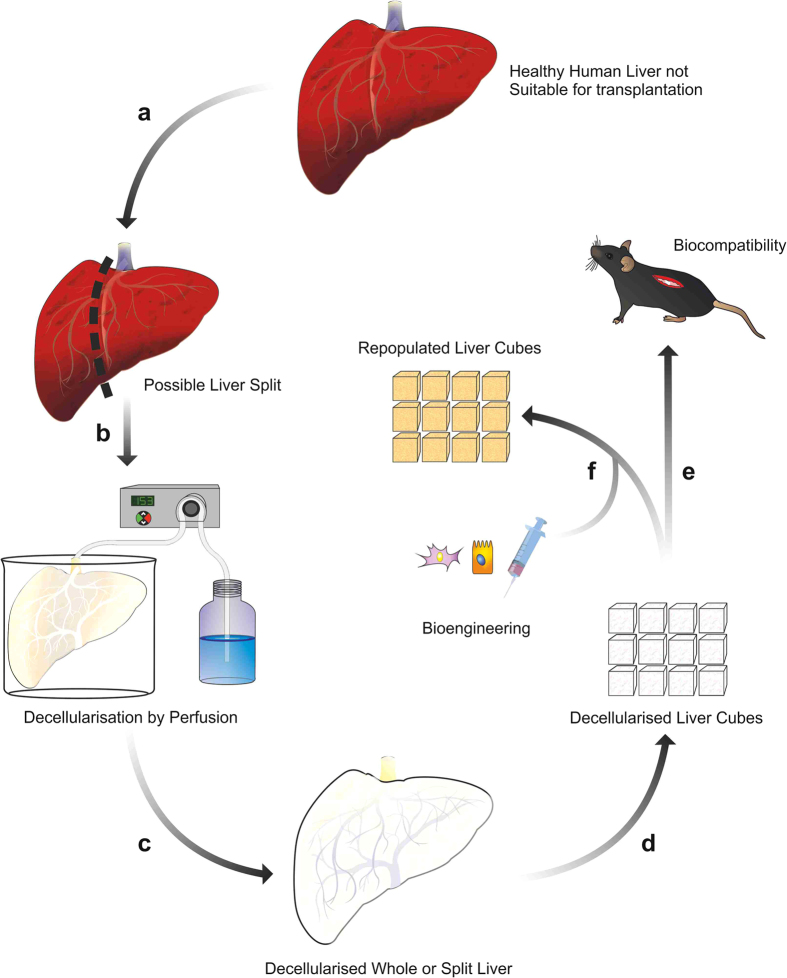
Schematic study plan. Human liver unsuitable for transplantation is surgically processed in order to obtain an isolated left lobe or is used as a whole (**a**,**b**). Lobes or whole organs are cannulated and decellularized by retrograde perfusion (**c**). Once decellularization is completed, human liver scaffolds are dissected by scalpel cleavage to obtain liver cubes (**d**) as a 3D-platform for biocompatibility and bioengineering studies (**e**,**f**).

**Table 1:** Perfusion protocol.

	Steps and Reagents				
Days	1^st^	2^nd^	3^rd^	4^th^	5^th^
−1	Thaw liver overnight at 4 °C				
0	dH_2_O	0.025% Trypsin-EDTA			
1	dH_2_O	0.01% SDS	0.1% SDS	1% SDS	
2	dH_2_O	0.025% Trypsin-EDTA	1% SDS		
3	dH_2_O	3% TX100			
4	dH_2_O	3% TX100			
5	dH_2_O	3% TX100			
6	dH_2_O	3% TX100	1X PBS		
7	dH_2_O	0.025% Trypsin-EDTA			
8	dH_2_O	1% SDS			
9	dH_2_O	1% SDS			
10	dH_2_O	1% SDS			
11	dH_2_O	1% SDS			
12	dH_2_O	1% SDS			
13	dH_2_O	PBS/AA 5%	3% TX100		
14	dH_2_O	PBS/AA 5%	dH_2_O	0.1%PAA/4% EtOH	1X PBS sterile

The decellularization of the whole human liver was achieved by repeating three times the procedure shown in the Table (the freezing/thawing step was performed just once).

Abbreviations: dH2O (distilled water), TX100 (Triton X100), SDS (sodium dodecyl sulfate), A-A 5% (Antibiotic and Antimycotic), PAA (paracetic acid) and EtOH (ethanol).
